# Trends in Testosterone Prescriptions for Older Men Enrolled in Commercial Insurance and Medicare Advantage

**DOI:** 10.1001/jamanetworkopen.2021.27349

**Published:** 2021-09-29

**Authors:** Alexander Everhart, Katrina Harper, Molly Moore Jeffery, Zachary Levin, Nancy E. Morden, Ashwini Sankar, Pinar Karaca-Mandic

**Affiliations:** 1 University of Minnesota School of Public Health, Minneapolis, Minnesota; 2 OptumLabs Visiting Fellow, Eden Prairie, Minnesota; 3 Technomics Research LLC, Medina, Minnesota; 4 Mayo Clinic, Rochester, Minnesota; 5 UnitedHealthcare, Minnetonka, Minnesota; 6 Dartmouth College, Hanover, New Hampshire; 7 Carlson School of Management, University of Minnesota, Minneapolis; 8 National Bureau of Economic Research, Cambridge, Massachusetts

## Abstract

This cohort study of health claims data examines trends in testosterone prescriptions for older men in the US following 2014 changes to the US Food and Drug Administration’s stance on testosterone’s effects and safety risks.

## Introduction

Studies have identified potential cardiovascular risks associated with testosterone therapy.^1^ In 2014, the US Food and Drug Administration (FDA) issued a safety communication regarding testosterone,^6^ and the Endocrine Society issued a statement describing potential risks for men with heart disease using testosterone. The following year, the FDA modified testosterone labeling to reflect that evidence only supports prescribing testosterone when treating hypogonadism unrelated to aging and that testosterone possibly increases stroke and heart attack risk.^2^

Morden and colleagues^2^ found testosterone receipt decreased among Medicare fee-for-service patients after these FDA actions, with minimal differences in trends between patients with and without coronary artery disease (CAD). However, it is unknown whether commercially insured patient receipt also declined or if declines differed among patients with other cardiovascular conditions. In this cohort study, we examined testosterone receipt trends among older men with commercial insurance or Medicare Advantage (MA), both with and without several relevant cardiovascular conditions.

## Methods

We identified patient-calendar-quarter observations for men aged over 50 years with at least 1 year of continuous medical and prescription insurance enrollment from 2007 to 2018 in the OptumLabs Data Warehouse (OLDW) claims database. OLDW is a longitudinal, real-world data asset with deidentified administrative claims data.^3^ From OLDW, we extracted patient age; evidence of CAD, arrhythmia, congestive heart failure (CHF), hypogonadism unrelated to aging (eg, Klinefelter syndrome, gonadal dysgenesis), and Elixhauser comorbidities^4^; and testosterone receipt. Testosterone receipt was considered indicated (“on-label”) if patients had 2 or more claims with a hypogonadism diagnosis unrelated to aging in the current or previous 4 quarters.

We used linear segmented models^5^ to regress current quarter testosterone receipt on age; number of Elixhauser comorbidities; nonmutually exclusive binary indicators for CAD, CHF, and arrhythmia; time trends with breaks following FDA actions; and cardiovascular condition–time trend interactions. We assessed whether trends differed by cardiovascular condition using Wald tests. Models were separately estimated by testosterone indication and insurance type.

This study analyzed preexisting deidentified data and was exempt from institutional review board approval as determined by the University of Minnesota institutional review board. This study followed Strengthening the Reporting of Observational Studies in Epidemiology (STROBE) reporting guideline for cohort studies. All tests of statistical significance were evaluated using 2-sided tests with a significance threshold of *P* < .05. All analyses were performed using STATA MP version 14.0 (StataCorp).

## Results

Our sample included 19 708 405 patient-calendar-quarter observations in MA and 25 254 984 observations in commercial insurance. Mean age (SD) was 72.8 (7.0) years in MA and 60.7 (6.5) years among commercial patients. In MA, 4 790 756 patient-quarters (24.3%) had CAD, 3 333 771 (16.9%) arrhythmia, and 1 862 576 (9.5%) CHF; among commercial patient-quarters, 2 398 979 (9.5%) had CAD, 1 459 332 (5.8%) arrhythmia, and 525 379 (2.1%) CHF.

**Figure 1.**
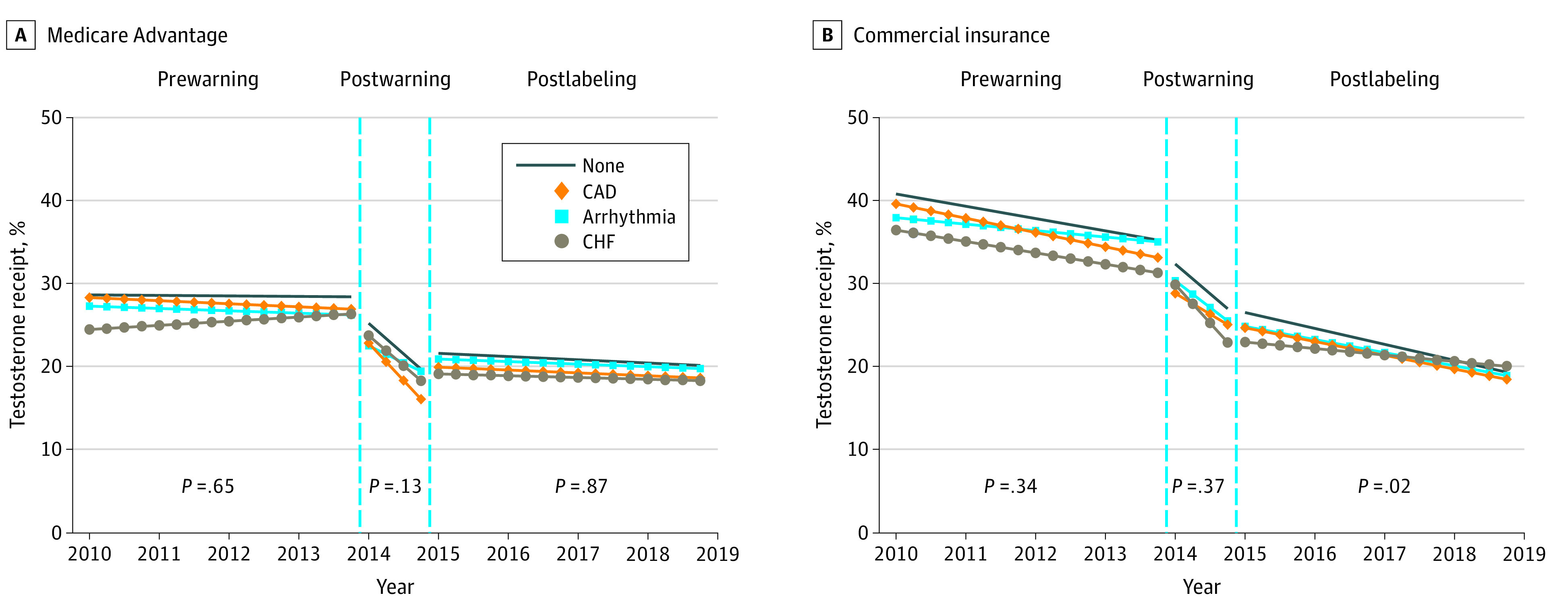
Adjusted Trends in On-Label Testosterone Receipt Trends in testosterone receipt adjusted for age and number of Elixhauser comorbidities. Observations were considered indicated for testosterone therapy (on-label) if they had 2 or more claims with a diagnosis of hypogonadism unrelated to aging in the current quarter or previous 4 quarters. Cardiovascular conditions identified based on presence of 2 or more claims with the given diagnosis in the current quarter or previous 4 quarters. *P* values for differences in trends derived from Wald statistics assessing whether interaction terms between relevant trend parameter and cardiovascular conditions are jointly equal to zero reported in callout box. Standard errors were clustered at the patient level. CAD indicates coronary artery disease; CHF, congestive heart failure.

A total of 574 789 MA patient-quarters (2.9%) and 916 404 commercial patient-quarters (3.6%) had hypogonadism unrelated to aging. This group had higher unadjusted testosterone receipt rates (MA, 128 504 patient-quarters [22.4%] vs commercial, 268 725 patient-quarters [29.3%]) than people without the condition (MA, 37 034 patient-quarters [0.2%] vs commercial, 117 874 patient-quarters [0.5%]). Adjusted receipt declined following the 2014 FDA and Endocrine Society actions and flattened after the 2015 labeling change. For example, patients in MA without relevant cardiovascular conditions had an adjusted receipt rate of 28.0% (95% CI, 26.9%-29.0%) in the last quarter of 2011, 19.1% (95% CI, 18.1%-20.1%) in the last quarter of 2014, and 19.5% (95% CI, 18.8%-20.2%) in the last quarter of 2018 (Figure 1).

**Figure 2.**
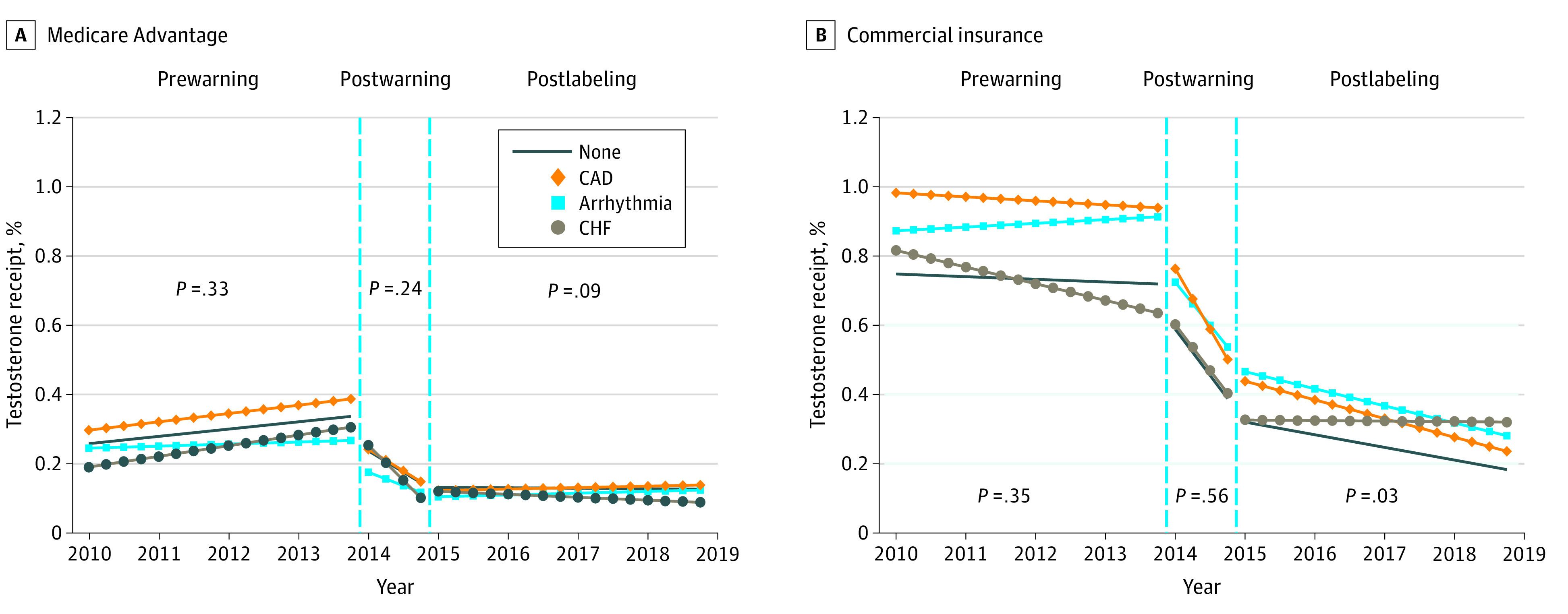
Adjusted Trends in Off-Label Testosterone Receipt Trends in testosterone receipt adjusted for age and number of Elixhauser comorbidities. Observations were considered not indicated for testosterone therapy (off-label) if they had fewer than 2 claims with a diagnosis of hypogonadism unrelated to aging in the current quarter or previous 4 quarters. Cardiovascular conditions identified based on presence of 2 or more claims with the given diagnosis in the current quarter or previous 4 quarters. *P* values for differences in trends derived from Wald statistics assessing whether interaction terms between relevant trend parameter and cardiovascular conditions are jointly equal to zero reported in callout box. Standard errors were clustered at the patient level. CAD indicates coronary artery disease; CHF, congestive heart failure.

MA receipt trends did not vary by cardiovascular condition. However, postaction trends in commercial on-label receipt differed by cardiovascular condition, ranging from a 0.194 (95% CI, −0.466 to −0.077) percentage point quarterly decline among patients with CHF to a 0.480 (95% CI, −0.527 to −0.433) percentage point quarterly decline among patients without cardiovascular conditions (Figure 1). Postaction off-label trends among commercial patients ranged from no change (95% CI, −0.007 to 0.007) among patients with CHF to a 0.014 (95% CI, −0.017 to −0.010) percentage point quarterly decline among patients with CAD (Figure 2).

## Discussion

Older men with MA and commercial insurance received testosterone less often following FDA actions. Our study was limited by its claims data: testosterone purchased without insurance was unobservable, and inaccurate claims coding could have caused misclassification of indications for testosterone.

Our results suggest potential for more nuanced testosterone prescribing. After warnings, on-label testosterone receipt decreased. Decreases in on-label and off-label receipt were not consistently associated with cardiovascular conditions identified in safety communications. Future studies should further explore responses to safety communications.
